# Screening blood donors for malaria, can we increase the number of eligible donors? An observational retrospective study

**DOI:** 10.1186/s12936-024-04966-3

**Published:** 2024-06-06

**Authors:** María Dolores Corbacho-Loarte, Oihane Martín, Sandra Chamorro-Tojeiro, Clara Crespillo-Andújar, Francesca F. Norman, José A. Pérez-Molina, Marta González Sanz, Marta Rosas Cancio-Suárez, Gabriel Ruiz-Calvo, Alberto Richart López, José Miguel Rubio, Rogelio López-Vélez, Begoña Monge-Maillo

**Affiliations:** 1 Infectious Diseases Department, National Referral Unit for Tropical Disease, Hospital Ramón y Cajal, IRYCIS, Madrid, Spain; 2 CIBER de Enfermedades Infecciosas, Instituto de Salud Carlos III, Madrid, Spain; 3 Microbiology Department, National Referral Unit for Tropical Disease, Hospital Ramón y Cajal, IRYCIS, Madrid, Spain; 4 Biostatistics Department, Hospital Ramón y Cajal. IRYCIS, Madrid, Spain; 5 Regional Transfusion Center, Madrid, Spain; 6 National Center for Microbiology, Instituto de Salud Carlos III, Madrid, Spain

**Keywords:** Transfusion, Malaria, *Plasmodium*, Asymptomatic infection, Immigrants

## Abstract

**Background:**

In non-endemic countries, malaria can be transmitted through blood donations from imported cases. To ensure standards of quality and safety of human blood, the European Union and Spanish national law, requires a deferral period, or a screening by immunological or genomic test among those donors with potential risk of malaria. Scientific societies, European Committee on Blood Transfusion, and Spanish Society of Haematology and Haemotherapy, refer only to the result of the immunological test.

**Methods:**

An observational retrospective study was performed in potential donors with a positive immunological test for malaria done in the Regional Transfusion Center in Madrid and referred to the National Reference Unit for Tropical Diseases in Madrid between 2015–2020. At consultation a Polymerase Chain Reaction (PCR) for malaria was performed.

**Results:**

During the study period, 121 possible donors attended for consultation at NRU-Trop. Median age: 38.5 (IQR:33–48); median time to consultation was 32 months (IQR:12.5–110). Eighty-two (67.8%) donors were migrants and thirty-nine were travellers (32.2%). ELISA values were available for 109 subjects (90.1%), 56 individual left malaria endemic area > 3 years before. All donors tested negative for *Plasmodium* spp PCR test (n = 121, 100%).

**Conclusions:**

None of the subjects with a positive immunologic test deferred as blood donors had a positive genomic test. The presence of *Plasmodium* spp in collected blood was not detected by molecular techniques. To avoid the loss of potential blood donors, especially those with low incidence red blood cell antigens, as more precise microbiology techniques become available, updating the existing legislation becomes necessary to increase the availability of donated blood.

## Background

Malaria is the parasitic disease with the highest associated morbidity and mortality worldwide. Although its incidence has decreased in the last decade, in 2021, near 247 million people from 84 endemic countries were diagnosed with malaria [1].

Although Europe is a non-endemic malaria area, 4856 malaria cases were reported in the European Union / European Economic Area (EU/EEA) in 2021, 4855 (> 99%) of which were confirmed cases. As many as 99.7% of the 4257 cases with known importation status were travel-related [2]. Although malaria transmission in non-endemic areas is rare, 11 cases were confirmed as acquired in the EU in 2021 (three in Greece and eight in France) [3, 4]. Also, several cases of congenital malaria and organ- and transfusion-transmitted malaria have been reported [5–7].

Regarding the transmission of malaria through transfusion, the EU/EEA has adopted four directives that regulate standards of quality and safety of human blood (2002/98/EC, 2004/33/EC, 2005/61/EC, and 2005/62/EC) [8]. In addition, the 2004/33/EC European Directive was adopted in the Spanish national legislation in 2005 (*Real Decreto* 1088/2005) [9, 10].

The European Directive and Spanish national law include specific sections about the information that should be required from donors to initiate of donation of blood components. Thus, a questionnaire is used to collect information about the health status and medical history; further information is collected through a personal interview performed by a qualified healthcare professional. The aim of this interview is to determine whether the donor may pose a health risk to others, due to the potential presence of transmissible diseases. (Part B-Annex II).

### Specifically for malaria infection, four possible scenarios are described in current legislation

- Donors with a history of previous malaria infection. Donation will be deferred for a period of three years following treatment completion. After this period, they will be accepted if they have a negative immunologic or molecular genomic test.
- Donors with a history of undiagnosed febrile illness during a visit to or within 6 months after departure from a malaria-endemic area. Donation will be deferred for a period of 3 years following the resolution of symptoms. This period may be reduced to 4 months if they have a negative immunologic or molecular genomic test.
- Asymptomatic donors who have lived in a malaria-endemic area within their first five years of life. Donation must be deferred for a period of 3 years following their return from the last visit to a malaria-endemic area. This period may be reduced to 4 months if they have a negative immunologic or molecular genomic test.
- Asymptomatic donors who have visited a malaria-endemic area. Donation must be deferred for a period of 6 months after departure from a malaria-endemic area unless they have a negative immunologic or molecular genomic test.

Therefore, malaria is an infectious disease that requires a deferral period, which may be reduced or eliminated if the donor’s immunologic or molecular genomic test is negative [9, 10]. However, the Guide to the Preparation, Use, and Quality Assurance of Blood Components of the European Committee on Blood Transfusion (EDQM, 21th Edition, 2023) and the Standards in Hemotherapy of the Spanish Society of Hematology and Hemotherapy (SEHH, 5th Edition, 2019), widely used in common clinical practice, only recommend the use of immunological tests for the screening of malaria in potential donors who have lived or visited an endemic malaria area [11, 12].

### Objective

The aim of the study was to describe the number of malaria cases confirmed by molecular testing among individuals deferred/rejected as possible donors due to a positive immunological test for *Plasmodium* spp.

## Methods

### Study design and participants

An observational retrospective study was performed at the National Reference Unit for Tropical Diseases (NRU-Trop) of the Ramón y Cajal University Hospital in Madrid, Spain, between January 2015 and December 2020.

All individuals were voluntary donors of the Regional Transfusion Center (RTC) in Madrid. Following the guidelines of scientific societies (EDQM and SEHH), all possible donors were asked about their country of origin, the countries they had lived in or visited, and the date of departure from a malaria endemic area. If there was a risk for malaria, immunological testing for *Plasmodium* infection was performed at the RTC. If positive, donors were deferred as donors and contacted from RTC and referred to the NRU-Trop or another Tropical Unit in Madrid. To evaluate a potential malaria infection, during this consultation at NRU-Trop, a new sample was collected to perform a Polymerase Chain Reaction (PCR) for *Plasmodium* spp. Deferred donors were classified into two groups: migrants (persons living in Spain born in an endemic malaria country) or travellers (Spanish travellers who had visited an endemic malaria area).

### Procedures

Demographic and epidemiological data included date of birth, age, gender, country of origin or visited country, date of consultation at the NRU-Trop, time from donation to consultation (defined as months elapsed from donation at regional transfusion center to consultation at the NRU-Trop) and time to consultation (defined as months elapsed from leaving a malaria-endemic country to consultation at NRU-Trop).

Screening for malaria was performed by serology. Qualitative and semi-quantitative detection of antibodies against *Plasmodium* spp (*Plasmodium falciparum*, *Plasmodium vivax*, *Plasmodium ovale* and *Plasmodium malariae*) in human serum or plasma was performed by Enzyme-Linked-Immunoassay (Bio-Rad®,France) [13] measuring absorbance A_450_. According to the manufacturer’s instructions, for assay validation, the negative should be lower or equal to 0.080. For the positive control sample to cutoff ratio (S/CO) should be greater than or equal to 1.000.

Confirmation of malaria infection was performed by PCR. During the study period, two different PCRs were used: until December 2017, an in-house Semiminested Multiplex-PCR [14] based on the amplification of small subunit of the human ribosomal (ssrRNA) with a detection limit of 0.01–0.001 parasites/µl and since January 2018, a commercial Real–Time Multiplex-PCR (Plasmodium Typing Real-Time PCR, Bio-Evolution®, France) with a detection limit of 10 copies/µl. This PCR detects genes coding for ssrRNA, AMA1 or Plasmepsin of *P. falciparum, P. malariae, P. ovale, P. vivax* and *P. knowlesi*. A region of human β-actin gene is targeted as internal control. Whole blood samples in EDTA tubes were used for PCR and stored at 4ºC for a maximun of 7 days prior to processing.

### Statistical analysis

Descriptive statistics were used to assess epidemiological characteristics. Continuous variables were presented as means with standard deviations and median values with their 25th-75th percentiles. Categorical variables were expressed as absolute frequencies and percentages. Normality tests were performed. The Mann–Whitney *U* test was used to examine potential associations between immunological test results and time to consultation. Time to consultation was divided into two categories (< 3 years or ≥ 3 years). Statistical analyses were performed using statistical package STATA®, Version 17.0 (StataCorp LP, College Station, TX, USA).

### Ethical statement

The patient database was approved by the Ethics Committee of Ramon y Cajal Hospital (179/14). All donors provided written informed consent.

## Results

During the study period, 121 possible donors attended for consultation at NRU-Trop. Sixty-four (52.9%) were men and median age was 40 years (38–41). Median time to consultation was 32 months (12.5–110).

**Fig. 1:**
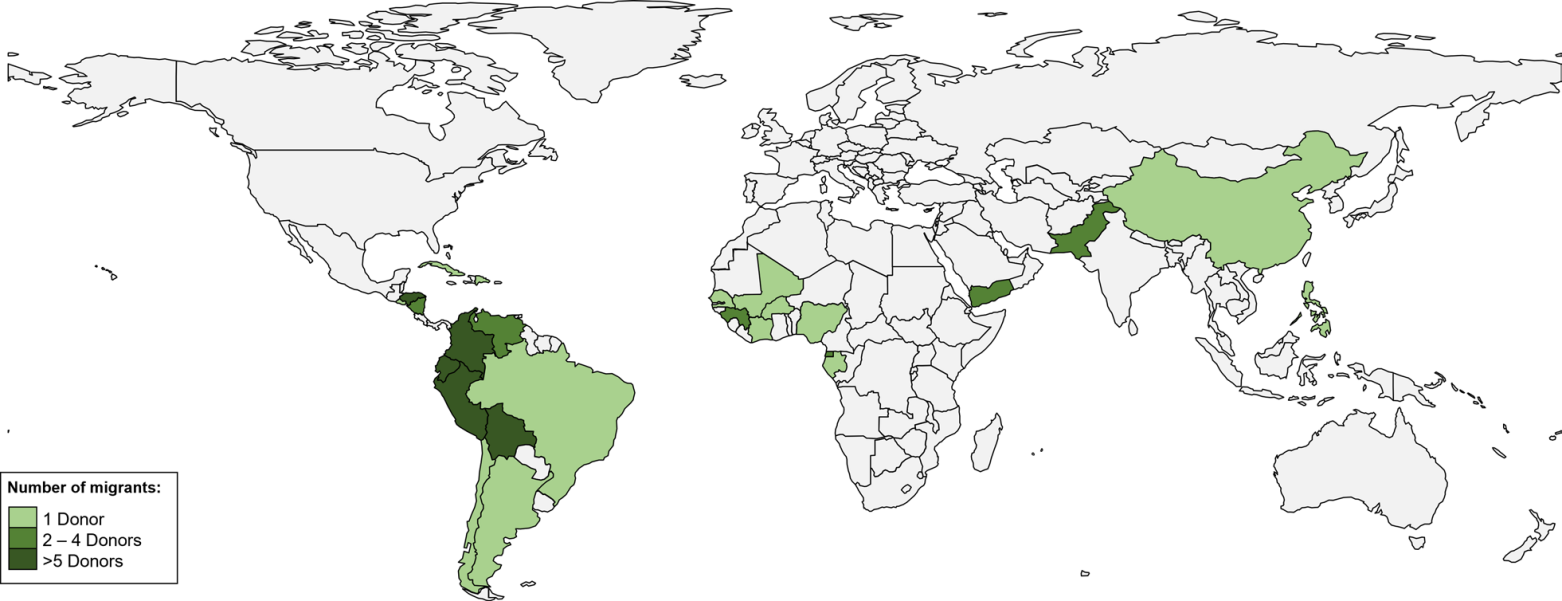
Migrants: Country of origins

Eighty-two (67.8%) donors were migrants, of whom 44 (53.7%) were men; the median age was 39 years (30–47) (Table 1). The main continent of origin were the Americas (n = 64, 78.1%), followed by Africa (n = 12, 14.6%) and Asia (n = 6, 7.3%). The most frequent countries of origin were Ecuador (n = 25, 30.5%) and Bolivia (n = 9, 10.9%) (Fig. 1). The median time from departure from a malaria-endemic area to consultation was 47.5 months (17–125). Specifically, 43 donors (52.4%) had left a malaria-endemic area more than 36 months before (3 years) and 22 donors (26.8%) had left a malaria-endemic area more than 120 months before (10 years). Two donors from Africa (Equatorial Guinea) had left an endemic malaria area more than 600 months before (> 50 years).

**Table 1:** Epidemiological characteristics of all potential donors

	Migrants	Travelers	Total
	n = 82	n = 39	n = 121
Age (years)	38.8 (30.4–46.6)	41.7 (34.8–49.4)	40 (38–42)
Gender			
Male	44 (53.7%)	20 (51.3%)	64 (52.9%)
Female	38 (46.3%)	19 (48.7%)	57 (47.1%)
Time to consultation (months)	47.5 (17–125)	18 (6–62)	32 (12.5–110)

**Fig. 2:**
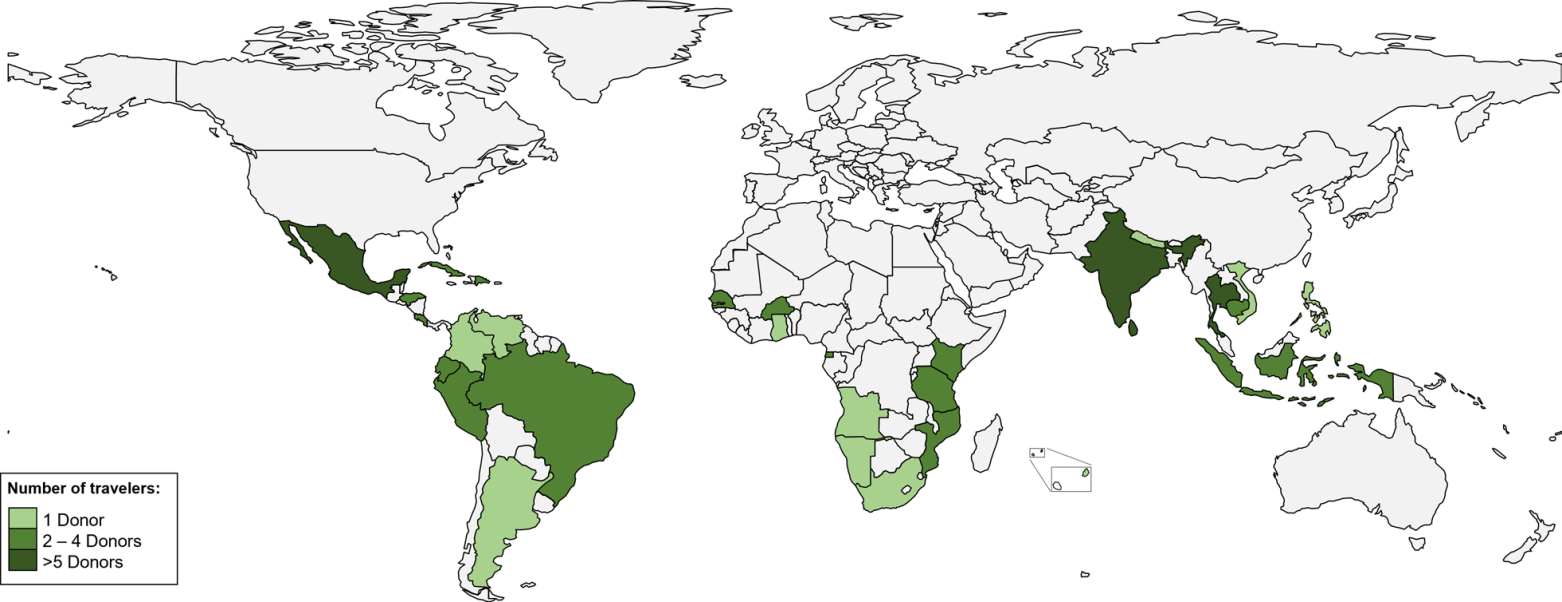
Travelers: Country visited

Thirty-nine (32.2%) donors were travellers. Among them 20 (51.3%) were men, and median age was 42 years (35–49). Twenty-three donors (58.9%) had travelled to an endemic area more than once, with a total of 78 travels. Sixteen donors (41.0%) had travelled to an endemic area once, 7 (17.5%) had travelled twice, and 16 (41.0%) had travelled to an endemic area three or more times. The most frequently visited area were the Americas, (n = 33, 42.3%), followed by Asia (n = 25, 32.1%) and Africa (n = 20, 25.6%). The most frequently visited countries were Mexico (n = 10, 12.8%), Thailand (n = 10, 12.8%) and India (n = 5, 6.4%) (Fig. 2). The median time from departure from a malaria-endemic area to consultation was 18 months (6–62).

ELISA was performed in all potential donors, but information regarding the value obtained was not available for all of them. In such cases, results were only informed as positive, without data on the value obtained. ELISA values were available for 109 subjects (90.1%). Since 60% of travellers had travelled to a malaria-endemic area more than once, ELISA values could not be related to the country visited. ELISA values for migrants are described by continent in Table 2.

**Table 2:** ELISA values for migrants

AREA	ELISA	Time to consultation (months)
America (n = 61)	3.99 (1.44–19.30)	57 (19.5–132)
Asia (n = 4)	6.85 (3.93–10.52)	61.5 (17–117)
Africa (n = 7)	7.28 (1.65–17.07)	18 (3–142.5)
TOTAL (n = 72)	3.45 (1.48–16.97)	47.5 (17–125)

**Fig. 3:**
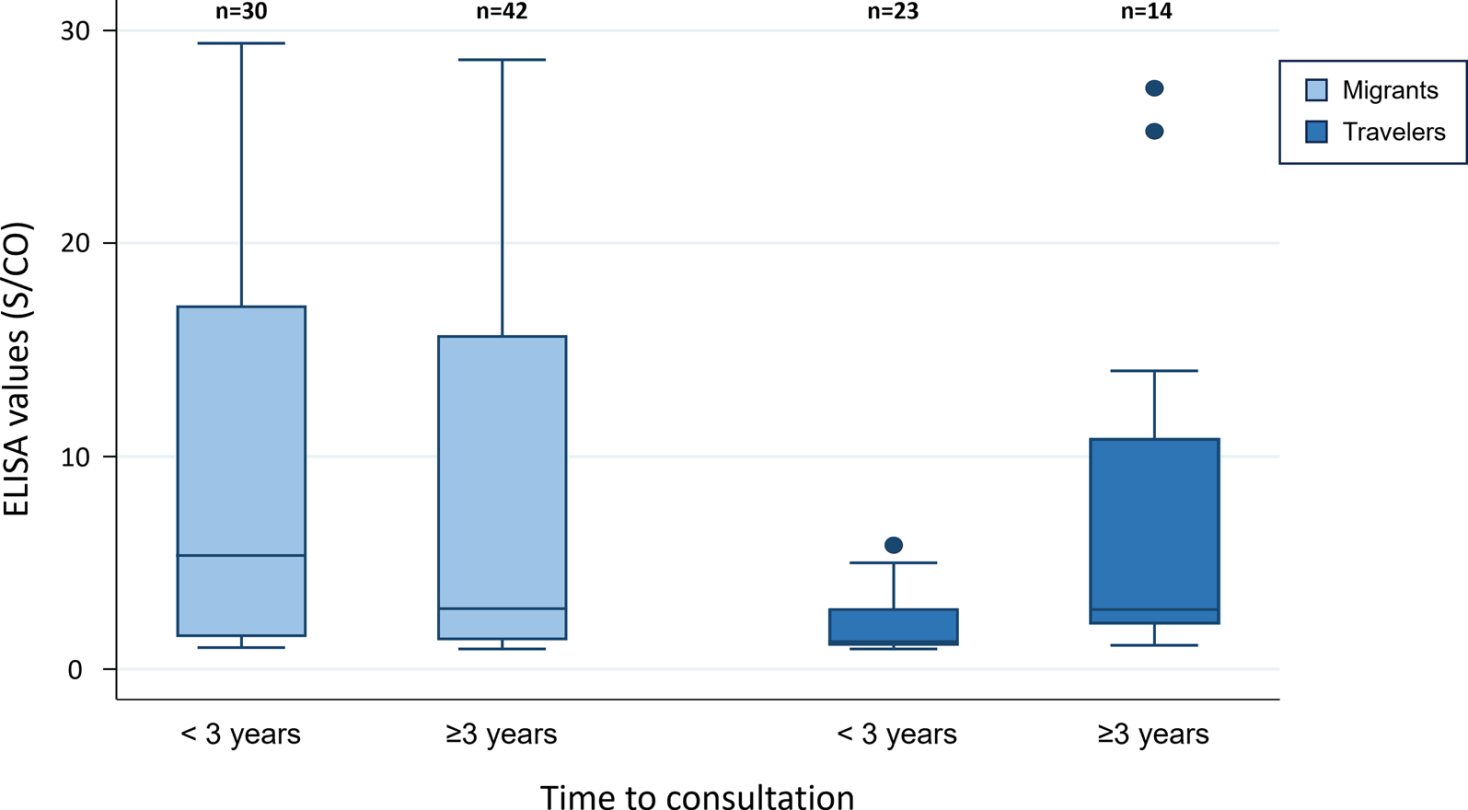
Boxplot: ELISA and time to consultation

Regarding ELISA values and time to consultation for migrants (n = 72, 66.1%), there were no significant differences in ELISA values based on whether time to consultation was more or less than three years (p = 0.545). In contrast, for travellers (n = 37, 77.9%) significant differences were found between both groups (p = 0.003) (Fig. 3).

At the NRU-Trop, all donors tested negative for the *Plasmodium* spp Seminested PCR (n = 61, 50.4%) and for the commercial PCR (n = 60, 49.6%).

## Discussion

In this study, *Plasmodium* RNA was not detected by genomic testing, a technique with a high sensitivity and specificity [15–17], in any of the 121 blood donors previously deferred for a positive antibody test.

Immunological/genomic testing is required by European and Spanish legislation in donors born in, living in, or visiting a malaria-endemic area. Subjects with a positive result are temporarily excluded as donors [9, 10]. However, the recommendations of scientific societies such as EDQM and SEHH only refer to the result of the immunological test and not recommend nucleic acid testing (NAT) for use in screening of blood donors [11, 12].

In this study, none of the subjects with a positive serological test had a positive molecular diagnostic test. These results may reasonably exclude an existing malaria infection and the presence of parasitaemia potentially transmissible by blood transfusion. However, regardless of the results obtained, none of these subjects were subsequently considered as donors because neither the European or Spanish legislation or guidelines specified what to do in these cases.

These results raise several questions. The first question is how to interpret the results of the malaria serology. A positive serologic result reflects that the donor has been potentially exposed to malaria antigens but does not necessarily indicate acute or asymptomatic malaria infection. Therefore, positive serology does not demonstrate the infectivity of blood components [18].

Moreover, it has been reported that some serological tests may have a low sensitivity for the detection of antibodies against *Plasmodium* antigens. A recent Italian study compared five ELISA donor screening kits, and sensitivity ranged from 53 to 64%. As a result, the same individual may be included or excluded from donation depending on the test used [19]. Additionally, a false positive result can be obtained due to a crossing reaction with other diseases [20, 21].

To avoid the loss of donations due to positive immunological positive tests for malaria antibodies, EDQM and SEHH recommend reevaluating the potential donor after a period of three years. However, this study shows that repeating tests is not effective, as immunological tests remained positive after three years in travellers, and migrants.

The other question is about the role of NAT in the screening of malaria in donors. Although genomic testing is mentioned in European and Spanish legislation, it is not referred to in EDQM or SEHH guidelines. From a microbiological point of view, molecular techniques are more accurate in screening for malaria infection. In fact, these techniques can detect 0.001–0.01 parasite per microlitre, detecting submicroscopic asymptomatic malaria infection that cannot be diagnosed by thick-smear and thin–film microscopy^15,16^. Molecular tests have been used in several studies for the screening of imported malaria infection in asymptomatic migrants, with prevalence being as high as 14.25%, being more frequent in those who have left malaria-endemic area ≤ 3 years before [22, 23]. Therefore, molecular testing seems to be the most accurate technique in detecting malaria infection with existing parasitaemia. Unlike serology, molecular testing will be negative in patients with past/treated malaria. Therefore, its use would allow more precise identification of possible malaria-transmitting donors. Furthermore, a recent study conducted in the USA presents automated assays that detect ribosomal RNA for routine donor testing with high sensitivity [24, 25].

The use of screening methods is of paramount importance to prevent donations of potentially malaria-transmitting blood. In this regard, serology appears to offer these guarantees. In fact, in a systematic review of transfusion-transmitted-malaria (TTM) in a non-endemic area, only 100 cases reported of TTM were reported and thirty-eight of these occurred in Europe [26].

The main challenge is the development of an optimal screening algorithm that ensures the safety and quality of blood components and at same time does not result in a loss of potential donors. The first step, which includes an initial structured questionnaire to assess the risk for malaria infection and subsequent immunological testing, without doubt should be maintained. However, new strategies should be explored to avoid the loss of potential donors with low incidence red cell antigens, which are rare in Caucasians but common in people from Asia, the Americas, or Latin America [27–29].

Kitchen et al*.* [30] carried out a study in the UK, with a recruitment period of almost three and a half years in which 140,000 potential donors had an epidemiological risk of malaria. Of these, 3.1% had a positive result in the serological test. In the study, these patients underwent a confirmatory technique (IFAT—Antibody Immuno-fluorescent Test), which was positive in only 0.84% of the total sample and 0.01% were positive by PCR. Extrapolating these data to our legislation, 3.1% of patients would have been rejected as donors [30].

With the increase in international travel to malaria-endemic regions, it is important to consider this donor profile. Moreover, the population of migrants from malaria-endemic areas is also increasing, and they are being denied the possibility of donating blood and as recipient, receive blood with low incidence red blood cell antigens.

Nonetheless, the study had several limitations. It is important to note that this is a retrospective study, with its associated limitations. This study describes a cohort of donors deferred for a positive immunological test, but no information about the total number of immunological tests performed is available. However, this study calls into question the correct implementation of the questionnaires that are given to donor and on which are decided to performed malaria screening. Based on these results, immunological testing was performed on 56 individuals, who had left a malaria-endemic area more than three years before, therefore, did not meet the epidemiologic criteria for determining the groups to be screened. This may affect screening results and highlights that immunological testing may not be clearly representative of malaria transmission risk.

## Conclusion

In potential donors with a positive immunological test, but asymptomatic and with low epidemiological risk factors, molecular testing should be considered. In this way, individuals with negative molecular test could be allowed as donors. However, to reduce human error, probably a second PCR determination or DNA extraction should be considered for ensure the safety of blood components. However, further research is required to determine the most appropriate screening algorithms for malaria, to avoid the loss of potential blood donors, but also to continue ensuring the quality and safety of blood components. In addition, as more precise microbiology techniques become available, updating the existing legislation becomes necessary to increase the availability of donated blood.

## Data Availability

Data is available on demand of corresponding author.
